# Unlocking Reishi’s secrets: nutritional and medicinal traits of *Ganoderma lucidum* isolated from tree bark in Egypt

**DOI:** 10.1186/s13568-025-01905-6

**Published:** 2025-07-12

**Authors:** Ramy M. Shady, Mona H. El-Gayar, Eman El-Gebaly, Hatem S. Abbass, Mahmoud A. Yassein

**Affiliations:** 1 Department of Microbiology and Immunology, Faculty of Pharmacy, October 6 University, 12585 Giza Egypt; 2 Department of Microbiology and Immunology, Faculty of Pharmacy, Ain Shams University, Organization of African Unity Street, Cairo, 11566 Egypt; 3 Department of Pharmacognosy and Medicinal Plants, Faculty of Pharmacy, Al-Azhar University (Boys), Cairo, 11884 Egypt; 4 Department of Pharmacognosy, Faculty of Pharmacy, Sinai University - Kantara Branch, Ismailia, 41636 Egypt

**Keywords:** *Ganoderma lucidum*, Water extract, Antitumor, Antimicrobial, Antiviral, Liquid chromatography-mass spectrometry

## Abstract

*Ganoderma lucidum* is an edible mushroom with high consumption rates in many cultures especially East Asian. This study assessed the *in-vitro* antimicrobial, antitumor, antiviral as well as antioxidant capacity of *Ganoderma lucidum* water extract (GLWE). Different microorganisms (standard strain bacteria and clinical isolates) were affected by mushroom aqueous extract, with inhibition zone diameters ranging between 16.1 and 38.8 mm. The MTT assay was used to evaluate antitumor efficacy against three distinct tumor cell lines: HepG-2 (liver tumor cell line), MCF-7 (breast tumor cell line), in addition to Caco2 (colorectal carcinoma cell line). GLWE was found to be safe for Vero cells (CC_50_ 5237 ± 1.6 µg/mL) and had detectable anticancer efficacy against all tested cell lines with IC_50_ ranges from 99.06 to 198.1 µg/mL. The Petricevich and Mendonça technique was used to test the antiviral activity against herpes simplex virus-1 together with Human adeno virus-7. The findings demonstrated modest antiviral activity, with EC₅₀ values of 493.8 ± 0.98 and 607.1 ± 1.9 µg/mL and corresponding selectivity indices of 10.6 and 8.62, respectively. On studying the antioxidant capacity of GLWE by using DPPH and ABTS, a potential activity with IC_50_ values of 23.69 ± 0.85 and 14.79 ± 0.92 µg /mL, respectively, were obtained. Liquid chromatography-mass spectrometry in addition to high-performance liquid chromatography analysis indicated a diverse range of compounds including Ganoderic acids, Lucidenic acids, Ganolucidic acids, and Ganoderenic acids which are characteristic triterpenoids to the genus Ganoderma, as well as some polyphenols and flavonoids. In addition, some water/fat soluble vitamins were also detected, as well as proteins and carbohydrates contributing to the high nutritional value of the studied mushroom.

**Supplementary Information:**

The online version contains supplementary material available at 10.1186/s13568-025-01905-6.

## Introduction

*Ganoderma lucidum*, commonly referred to as reishi or lingzhi, is a macrofungus belonging to the basidiomycete group that has been consumed in traditional medicinal practices for millennia, particularly within East Asian cultures (Hapuarachchi et al. 2015). Morphologically, this species is delineated by a lustrous, reddish-brown, kidney-shaped cap exhibiting a lacquer-like appearance and a robust, woody texture (Kapoor and Sharma 2014). The fruiting body generally displays concentric rings and may attain a diameter of up to 20 cm. This basidiomycete fungus is classified within the family Ganodermataceae and reproduces via brown, double-walled spores (Sudheer et al. 2018). *G. lucidum* naturally thrives in warm and humid forest ecosystems and is frequently observed proliferating on decomposing hardwood trees, specifically oaks, maples, and elms. It exhibits a preference for subtropical to temperate climatic conditions and is extensively distributed throughout Asia, North America, and select regions of Europe (González et al. 2020). Its capacity to colonize necrotic or senescent trees, in conjunction with its abundant secondary metabolite profile renders it a significant focus of pharmacological and biotechnological inquiry. Renowned for its wide array of therapeutic applications, *G. lucidum* is rich in bioactive compounds that underpin its anti-inflammatory, antioxidant, and immunomodulatory properties. Key constituents of *G. lucidum* include triterpenoids, polysaccharides, and phenolic compounds (Naim 2024). Recent studies emphasize the necessity of investigating the chemical composition and bioactivity of *G. lucidum* cultivated in diverse geographical regions, including Egypt, to assess the influence of local environmental factors on its medicinal efficacy (Oke et al. 2022).

Chemical profiling of *G. lucidum* is essential for elucidating the relationship between its bioactive constituents and their therapeutic activities. For the identification and measurement of complex phytochemical combinations, sophisticated analytical methods like High-Performance Liquid Chromatography (HPLC) along with Liquid Chromatography-Mass Spectrometry (LC–MS) have emerged as a substantial analytical tool in modern research (Qin et al. 2024). These methodologies enable a comprehensive analysis of the secondary metabolites present in *G. lucidum*, providing significant revelations regarding their potential health advantages and pharmacological characteristics. Although the mushroom's importance is well known, there is still a significant gap in research on the constituent profile of *G. lucidum* extracts from Egyptian sources.

Apart from its varied chemical makeup, *G. lucidum* has been demonstrated to exhibit a diverse array of bioactivities, including antiviral, antifungal, antioxidative, antibacterial, as well as anticancer effects (Wu et al. 2024). The capability of antioxidant substances to abolish free radicals and mitigate oxidative stress, is a significant determinant in mitigating the emergence of many chronic diseases making them especially significant (Muscolo et al. 2024). The antibacterial activities of *G. lucidum* have also garnered a lot of attention as possible substitutes for traditional antibiotics, particularly given the rising problem of antibiotic resistance (Erbiai et al. 2023).

Studying the bioactivities alongside the chemical composition of *G. lucidum* from Egypt could provide valuable insights into its potential applications in modern medicine. The findings could lay the groundwork for future studies and the use of this mushroom to support health and well-being.

Edible mushrooms are considered as nutraceuticals and functional nourishment due to their comprehensive nutritional composition and the concomitant health benefits they confer (Lesa et al. 2022). *G. lucidum* contains variable concentrations of carbohydrates and crude fiber, proteins and amino acids, lipids, minerals, as well as water and fat-soluble vitamins (El Sheikha 2022).

This study aims to evaluate biological activity, such as antimicrobial, anticancer, antiviral and antioxidant activity, and the nutritional potential of GLWE. Additionally, a thorough chemical analysis of the bioactive constituents found within the extract of the investigated fungal strain, isolated from Egypt, will also be performed.

## Materials and methods

### Sample collection

Morphologically preidentified mature *G. lucidum* grown on old wood logs and agricultural waste products were gathered from several locations in Kafr El sheikh governorate, Egypt (31°12′38.1"N 31°01′09.2"E) in December 2023. Sterile surgical blades were utilized to cut the fruiting body under aseptic conditions, afterwards stored in airtight sterile plastic bags before being taken to the lab for testing.

## Molecular identification of the collected fungal isolate

### Extraction of DNA and polymerase chain reaction (DNA amplification)

Total genomic DNA was extracted from the powdered dry fruiting body of the mushroom utilizing the E.Z.N.A.® Fungal DNA Mini Kit (Omega Bio-tek, Norcoss, Georgia, U.S) in accordance with the manufacturer’s guidelines. Specific gene amplification was performed using Dream Taq Green PCR Master Mix (2X) (Thermo Fisher Scientific, Massachusetts, United States) following the manufacturer’s guidelines, with amplification carried out on a Creacon Polymerase Chain Reaction (PCR) system (Creacon Technologies Ltd., Netherlands). The Internal transcribed spacer (ITS) region was utilized as a molecular identification marker. The primers ITS1 (TCCGTAGGTGAACCTGCGG) as well as ITS4 (TCCTCCGCTTATTGATATGC) were utilized (Park et al. 2012). The thermal cycler program was configured as outlined by Park et al. (2012), with the following parameters: 5 min of initial denaturation at 94 °C, 30 cycles of 30 s at 94 °C, 30 s at 56 °C, and 1 min at 72 °C, and a final 5 min of 72 °C extension time.

The PCR products were visualized using gel electrophoresis, carried out with a 1.5% agarose gel prepared in Tris–borate-EDTA buffer (pH 8.3) and supplemented with 0.5 µg/mL ethidium bromide. The ethidium bromide-stained amplicons were visualized under an ultraviolet transilluminator.

### DNA sequencing and internal transcribed spacer sequence analysis

The data analysis was conducted using the gel documentation system (Gel doc-it™ Imaging System, Cambridge, United Kingdom) and total lab analysis software Version 1.0.1. Positive amplicons of approximately 1500 base pairs were excised and eluted from the agarose gel. The final PCR products were quantified spectrophotometrically and purified using micro spin filters. Following the manufacturer’s guidelines, the pure PCR products were subjected to Sanger sequencing using the ABI PRISM® 3100XL Genetic Analyzer (Applied Biosystems, California, United states) with the specific β-tubulin marker genes ITS-1 (forward) as well as ITS-4 (reverse). Using the BLAST algorithm and NCBI reference sequences, the sequence similarity between produced and published sequences was assessed and a phylogenetic tree was constructed using NCBI tools and MEGA 12.0 software.

### Mushroom aqueous extract preparation

The dried fruiting bodies of the mushroom isolate were ground into a fine powder using a hammer mill, yielding 500 g of powdered material. The powder was then subjected to maceration in seven liters of distilled water at 25 °C for three consecutive days, repeating this process three times. After each maceration, the mixture was filtered and the collected filtrates (500 mL) were concentrated using a rotary evaporator (Rotavapor® R-100, BUCHI, Flawil, Switzerland) at 50 °C, yielding approximately 22 g of dry extract residue. Sterile distilled water was then used to dissolve the dried GLWE for preparation of various mushroom concentrations used in biological activities.

### Evaluation of the biological activity of GLWE

#### Cell lines and cultures

Vero cells were acquired from VACSERA in Agouza, Giza, Egypt. The used tumor cell lines include Hep-G2, MCF-7, and Caco2, were sourced from the Faculty of Medicine at Kasr Al-Ainy, Cairo, Egypt. Dulbecco's modified Eagle’s medium (DMEM; GE Healthcare Bio-Sciences, Pennsylvania, U.S) was supplemented with 10% heat inactivated fetal calf serum (Hyclone Laboratories Inc, Logan, UT 84321, U.S), 10 μg/mL of insulin (Sigma, Massachusetts, U.S), 100 μg/mL of streptomycin, alongside 100 U/mL of penicillin (GIBCO, Gaithersburg, MD) for cell line cultivation. A humidified environment with 5% carbon dioxide was used for incubation at 37 °C.

#### Cytotoxic effect against Vero cell line

Cytotoxicity was evaluated by means of the MTT (3-[4,5-dimethylthiazol-2-yl]-2,5 diphenyl tetrazolium bromide) assay. Cells were incubated for 24 h after the described medium was applied to 96-well plates that included 100 μL aliquots of cell suspensions (with a cell density of 1.2–1.8 × 10^4^ cells/well). Afterward, the cells were exposed to different concentrations of fungal extract for 48 h. These wells were then filled with MTT in phosphate buffer saline and incubated for four hours. After that, formazan crystals were dissolved in MTT solubilizing solution (anhydrous isopropyl alcohol containing 0.1 N hydrochloric acid and 10% Triton X-100, Sigma, Milwaukee, WI, USA), and a microtiter plate reader was utilized to determine the absorbance at 570 nm (El Sayed et al. 2016).

#### Antiviral effect

Herpes simplex virus type 1 (HSV-1) and human adenovirus type 7 (HAdv7), both procured from VACSERA in Agouza, Giza, Egypt. Following the methods described by Petricevich & Mendonça, (2003), the experimental protocol was carried out. Vero cells at a density of 10^5^ cells/mL were seeded in microtiter plates. Upon reaching confluency, growth media was drained, then cells were treated for 24 h at 37 °C in 5% CO_2_ with twofold serially diluted non-toxic concentrations of GLWE ranging from 15.62 to 2000 μg/mL (100 μL/well). Concurrently, control cell culture plates were maintained without treatment to serve as a baseline for viral control titration. Eagle's minimum essential medium (EMEM) was used to serially dilute the HAdv-7 virus and HSV-1 tenfold to reach 50% cell culture infectious dose (CCID50). Following the removal of the growth medium, viral dilution (0.1 mL/well) was inoculated into the Vero cell monolayers, regardless of whether the cells had been treated with the test extract or not. Wells that were left untreated and uninfected (mock-treated) served as negative controls. Acyclovir and cidofovir (at a concentration range 0.7—90 μg/mL) were employed as positive controls for HSV-1 and Adv7, respectively. The 96-well plates were kept at 37 °C in 5% CO_2_ for 3 days and observed daily using an inverted microscope. After complete destruction of the virus-infected cells, 20μL of MTT solution (7.5 mg/ml PBS) was added to each well of the microtiter plates. The plates were then incubated at 37 °C for 4 h. Approximately 100 μL of MTT solubilizing solution (anhydrous isopropyl alcohol containing 0.1 N hydrochloric acid and 10% Triton X-100, Sigma, Milwaukee, WI, USA) was added to each well to dissolve the formazan crystals. After shaking the plates for 10 min, a microtiter plate reader was used to determine the absorbance at 570 nm (Sudo et al. 1995). The 50% antiviral effective concentration (EC_50_) was expressed as the concentration that achieved 50% protection of virus-infected cells. The percentage protection was calculated by the following formula:

$$ \% {\mkern 1mu} protection{\mkern 1mu} = {\raise0.7ex\hbox{${\left[ {\left( {ODt} \right){\mkern 1mu} virus - \left( {ODc} \right){\mkern 1mu} virus} \right]}$} \!\mathord{\left/ {\vphantom {{\left[ {\left( {ODt} \right){\mkern 1mu} virus - \left( {ODc} \right){\mkern 1mu} virus} \right]} {\left[ {\left( {ODc} \right){\mkern 1mu} mock - \left( {ODc} \right){\mkern 1mu} virus} \right]}}}\right.\kern-\nulldelimiterspace} \!\lower0.7ex\hbox{${\left[ {\left( {ODc} \right){\mkern 1mu} mock - \left( {ODc} \right){\mkern 1mu} virus} \right]}$}} \times 100 $$

where (ODt)virus is the absorbance of the test extract, (ODc)virus is the absorbance of the virus-infected control (no GLWE), and (ODc)mock is the absorbances of the mock-infected control.

### Cytotoxic effect against tumor cell lines

The cytotoxicity of the GLWE was further assessed against selected cancer cell lines using the PromoKine Cell Proliferation Assay Kit IV (MTT). The cells were cultured in microtiter plates using DMEM supplemented with 10% heat inactivated fetal bovine serum and 1% antibiotic solution (penicillin-G and streptomycin). The 96-well plates were incubated for 24 h at 37 °C with 5% CO₂. To ensure proper population densities, cells were seeded with varying doses of the extract at densities ranging from 5,000 to 10,000 cells per well. After being washed twice, the final culture volume in each well was fixed at 0.1 mL. Subsequently, 10 μL of MTT solution was added to each well, and the plate was lightly tapped or shaken on an orbital shaker. After four hours of incubation at 37 °C, formazan salts formed by metabolically active cells were solubilized by 200 μL of DMSO solvent added to each well. The solution was pipetted several times to ensure complete mixing, reaching a final volume of 300 μL. Doxorubicin (Sigma-Aldrich, Dorset, UK) was used as a positive control. Absorbance was measured at 570 nm. Utilizing the Graph Pad prism 8.0.1, IC_50_ values, which indicate the concentration needed for 50% inhibition of cell viability, were deduced from the dose–response curves (Fig. S1). In addition, cellular morphologies of treated and untreated cells were compared using an inverted microscope (Nikon TMS, USA) fitted with a digital camera and analyzed with Cell Sense software (Yuce et al. 2023).

### Antibacterial and antifungal activity

A total of 39 bacterial strains of which 3 Gram positive standard strains (methicillin resistant *Staphylococcus aureus* (MRSA) ATCC 25923, *Enterococcus faecalis* ATCC 19433 and *Staphylococcus aureus* ATCC 43306) and 6 Gram negative standard strains (*Shigella flexneri* ATCC 12022, *Shigella Sonnie* ATCC 25931, *Proteus vulgaris* ATCC 49132, *Klebsiella pneumonia* ATCC 700603, *Pseudomonas aeruginosa* ATCC 27853 and *E. coli* ATCC 25922) and 4 fungal strains were also tested. Bacterial strains were provided by the Microbiology Department, Faculty of pharmacy, Ain shams university, Cairo, Egypt. In addition to six clinical isolates of *E. coli* as well as eight clinical isolates of MRSA, ten clinical isolates of *P. aeruginosa* and six multidrug-resistant (MDR) strains of *Klebsiella pneumoniae* were collected. This was alongside two unicellular fungal strains (*Candida albicans* ATCC 10231 and *Candida tropicalis* ATCC 13803) and two filamentous fungi (*Aspergillus niger* RCMB 002005 and *Penicillium marneffei* RCMB 001022), which were provided by Al-Azhar University's Regional Center of Mycology & Biotechnology (RCMB) in Cairo, Egypt. The standardized cup-diffusion technique, as illustrated by the Clinical & Laboratory Standards Institute (CLSI 2024), was carried out for conducting antimicrobial susceptibility testing.

The antimicrobial efficacy of GLWE (50 mg/mL) was assessed by measuring the inhibition zones (mm) encircling the wells. The antibiotic Ciprofloxacin (8 μg/mL) (MAST Diagnostics Ltd., Bootle, Merseyside, UK) and the antifungal ketoconazole (100 μg/mL) (Oxoid, UK) were used as positive controls.

### MIC assessment using a resazurin-based colorimetric method

A resazurin-based colorimetric technique was employed to assess the minimum inhibitory concentrations (MICs) of GLWE and its antibacterial activity. To prepare the resazurin solution, 337.5 mg of resazurin powder (BDH Chemicals Ltd, England) was solubilized in 50 ml of sterile distilled water, mixed for an hour, and stored in an amber glass container to prevent light exposure. The MIC was assessed using broth microdilution according to CLSI (2024) protocols. A microtiter plate was set up with controls for broth sterility, GLWE sterility, and growth, along with test wells for GLWE at various concentrations. Each well contained 100 μL of Mueller–Hinton broth, with the first well in each test column receiving 100 μL of sterile fungal extract, followed by a twofold serial dilution. A bacterial suspension (1.5 × 10^6^ cells/mL) was added to all wells except controls and mixed. After overnight incubation at 37 °C, 5 μL of resazurin dye was then added to all wells, followed by an additional four hours of incubation at 37 °C. The MIC was identified as the lowest concentration of GLWE before a color change occurred, indicating bacterial growth inhibition (Teh et al. 2017).

## Antioxidant activity

### DPPH scavenging capacity assay

The quantitative spectrophotometric experiment was performed with 0.1 mmol solution of 2,2`-Diphenyl-1-picrylhydrazyl (DPPH) dissolved in methyl alcohol. Using the previously elucidated approach, the IC_50_ value was utilized to compare the DPPH of the GLWE with that of ascorbic acid (Sigma, Burlington, MA, US) as a standard (Fig. S2) (El-Hela et al. 2020). The capacity to eliminate DPPH radical was illustrated utilizing the subsequent equation after the absorbance was measured at 517 nm.

$$ {\text{DPPHscavenging effect }}\left( \% \right)\, = \,\left[ {\left( {Acontrol - Asample} \right)/Acontrol} \right]\, \times \,100 $$

The concentration needed to lower the initial DPPH concentration by 50% was calculated using the IC_50_ values.

### ABTS radical cation scavenging capacity assay

The IC_50_ value was utilized to compare 2,2′- Azino-bis-3-ethylbenzothiazoline-6-sulfonic acid** (**ABTS) radical scavenging capacity of GLWE with vitamin C (Sigma, Burlington, MA, US) standard (Fig. S3) in accordance with the previously described method (El-Sherbiny et al. 2024). At 734 nm, the absorbance was measured by means of a UV/VIS spectrophotometer (Infitek Co., Ltd, China). ABTS radical scavenging capacity was calculated using the subsequent equation:

$$ ABTS \, scavenging \, effect \, \left( \% \right) \, = \, \left[ {\left( {A \, control - A \, sample} \right)/A \, control} \right] \, x100 $$

The IC_50_ values were calculated, which show the concentration needed to cut the initial concentration of ABTS radicals by 50%.

## Biochemical characterization of the GLWE

### Nutritional contents

#### Total carbohydrate content

The GLWE's total content of carbohydrate was assessed utilizing the phenol–sulfuric acid methodology, as illustrated by Rašeta et al. (2024). A standard calibration curve was constructed using glucose (Fig. S4A), with measured absorbances at 490 nm. The findings were signified as glucose equivalent in milligrams per gram of dry weight (mg GluE/g). The principle of this method relies on those carbohydrates, when exposed to strong acid and heat, produce furan derivatives. These derivatives subsequently condense with phenol to form stabilized golden yellow complexes, which can be quantified spectrophotometrically (Nielsen 2024). The assay was conducted using 1 mL of 5% phenol, and 5 mL of 96% sulfuric acid, alongside a serial dilution of standard glucose.

#### Vitamin content

A reversed-phase high-performance liquid chromatography (RP-HPLC) Column (5 µm, 250 × 4.6 mm) from SHIMADZU (Columbia, Maryland, U.S) was used to evaluate vitamins. Methanol (solution A) and 0.023 molar H_3_PO_4_ (pH 3.54) (solution B) made up the mobile phase. Vitamins were separated using isocratic elution (33/67-A/B), with a solvent flow rate of 1 mL /min. Water-soluble vitamins were identified using a diode array detector at 270 nm (Marzougui et al. 2009), while vitamins A, D, E, and K were detected at 325 nm, 265 nm, 290 nm, and 244 nm, respectively (Sami et al. 2014).

#### Proteins content

According to the conventional procedure, the Bradford protein assay was utilized to measure the protein content (Yang et al. 2021). This technique relies on Coomassie Brilliant Blue G-250 (CBBG-250) dye's ability to attach to proteins. By measuring the blue-colored solution's absorbance at 595 nm and comparing it to a standard curve (Fig. S4B) created using known quantities of the standard protein bovine serum albumin (BSA) in complex form, such as BSA-CBBG-250, the quantity of protein is ascertained (Bradford 1976). The results are determined as mg BSAE/g, or mg BSA equivalent per gram of dry weight.

## Functional phytoconstituents characterization and quantification

### Total content of phenolics

The Folin–Ciocalteu colorimetric technique illustrated by Singleton & Rossi. (1965) was used for calculating total phenolics content. In short, 3 mL of 10% Folin–Ciocalteau was combined with 5 μL GLWE and 800 μL of 7.5% NaHCO₃. The reaction solution was incubated at 25 °C for 30 min. The measured absorbance of the mixture was recorded at 765 nm using a Milton Roy Spectronic 1201 UV/Vis Spectrophotometer (Houston, Texas, USA). A calibration curve was created utilizing different gallic acid concentrations (Fig. S4C). The findings were signified as Gallic acid equivalent in mg per gram of dry weight (mg GAE/g) extract to express total phenolics content (Vl 1999).

### Total content of flavonoids

The Chang et al. (2002) technique was utilized to quantify the Total flavonoid content. Simply short, 300 μL of a five percent sodium nitrite solution as well as 3.9 mL of distilled water were combined with 100 μL of GLWE, and the mixture was left to react for 5 min. After that, 300 μL of a 10% AlCl_3_ solution was added, and the combination was left to continue to react for 6 min. Following this period, 2 ml of NaOH (1 mM^−1^) were added to the mixed solution. All samples were then mixed with 2.4 ml of distilled water. The Milton Roy Spectronic 1201 UV/Vis Spectrophotometer (Houston, Texas, United States) was utilized to measure the absorbance at 510 nm, with the sample blank as a reference. Varying concentrations of standard quercetin were used to create a calibration curve (Fig. S4D). The Total content of flavonoids in GLWE was represented as (mg QE/g).

### HPLC quantification of polyphenols and flavonoids

To identify polyphenol and flavonoid contents, the phytochemical makeup of GLWE was further assessed using the HPLC UV–Vis detector. Waters® Alliance 2690 HPLC system (Waters Ltd, Massachusetts, United States) Separations Module alongside 996 photodiode array detectors, as well as Inertsil ODS-3 HPLC Column, 5 μm, 250 × 4.6 mm (Reversed phase mode), was used for analytical purposes. Using external standards (Fig. S5), the phenolic and flavonoid components were detected at 280 nm. Seven flavonoid compounds (catechin, daidzein, hesperetin, kaempferol, naringenin, quercetin, and rutin) alongside eleven phenolic compounds (chlorogenic acid, cinnamic acid, caffeic acid, coumaric acid, ellagic acid, ferulic acid, gallic acid, methyl gallate, rosmarinic acid, syringic acid, and vanillic acid) make up the working standard solution. Methanol served as the mobile phase in a gradient separation process using buffer (0.1% H_3_PO_4_ in water) and a flow rate of 1 mL/min (Singh et al. 2017).

### LC–MS analysis for triterpenoids

A XEVO TQD triple quadruple mass spectrometer (Milford, CT, USA) having a column (50 mm × 2.1 mm ID × 1.7 μm particle size) running at a flow rate of 0.2 mL/min was used to perform electrospray ionization mass spectrometry (ESI–MS) in negative ion acquisition mode (Salem et al. 2024). The capillary and cone voltages had been adjusted to 3 kV and 30 V, respectively, as part of the instrument’s unique mass configuration. Desolvation temperature was set to 400 °C. 50 L/hr and 900 L/hr were the flow rates for cone gas and desolvation gas, respectively. Collisions with initial collision energies of 4 V, followed by 20 V, then 30 V, were used in the survey scan method. Resolutions of 10.3, 10.0, 14.8, and 15.1 were assigned to LM1, LM2, HM1, and HM2. The two solvents that comprised the binary mobile phase were solvent A (H₂O) and solvent B (C₂H₃N), both containing 0.1% CH₂O₂. Elution gradients involved an increase in solvent B from 10% (0 to 5 min) then 30% (5 to 15 min) then 90% (15 to 25 min) then 100% (25 to 29 min) then decreasing to 10% as a final composition which was kept constant to 32 min for re-equilibration. Masslynx software was used to process the analytical data. (Huang et al. 2024).

### Statistical analysis

For all conducted experiments, three replicates of each measurement were performed, and the findings are expressed as means ± standard deviation (SD). The data was processed by means of GraphPad Prism version 8.0.1 (GraphPad Software Inc., California, United States). Statistical comparisons between 2 independent groups were conducted by multiple paired t-tests with Holm–Sidak correction, whereas two-way ANOVA was employed for analyses involving two independent variables, followed by Bonferroni’s post hoc test for multiple comparisons. A p-value of < 0.05 was considered statistically significant.

## Results

### Molecular identification of mushroom

**Fig. 1:**
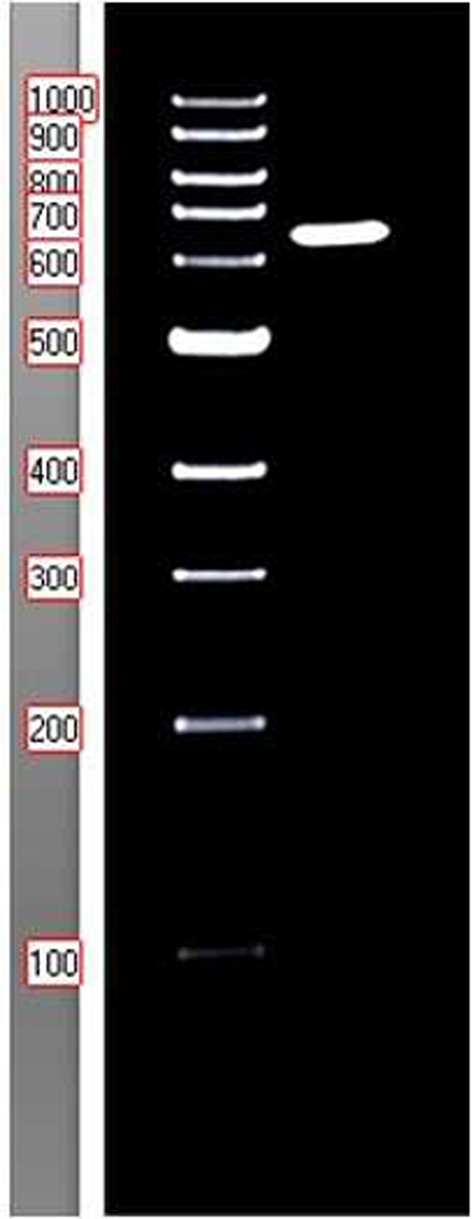
Molecular weight detection of amplicons of the nuclear ribosomal ITS region, with isolates showing an approximate size of 655 bp

The fungal DNA fragment sizes were ascertained by comparing them to a 100 base-pair DNA ladder (Fig. 1 and Table S1). (peqGOLD DNA-Ladder, Peqlab, VWR, Radnor, Pennsylvania, USA).

**Fig. 2:**
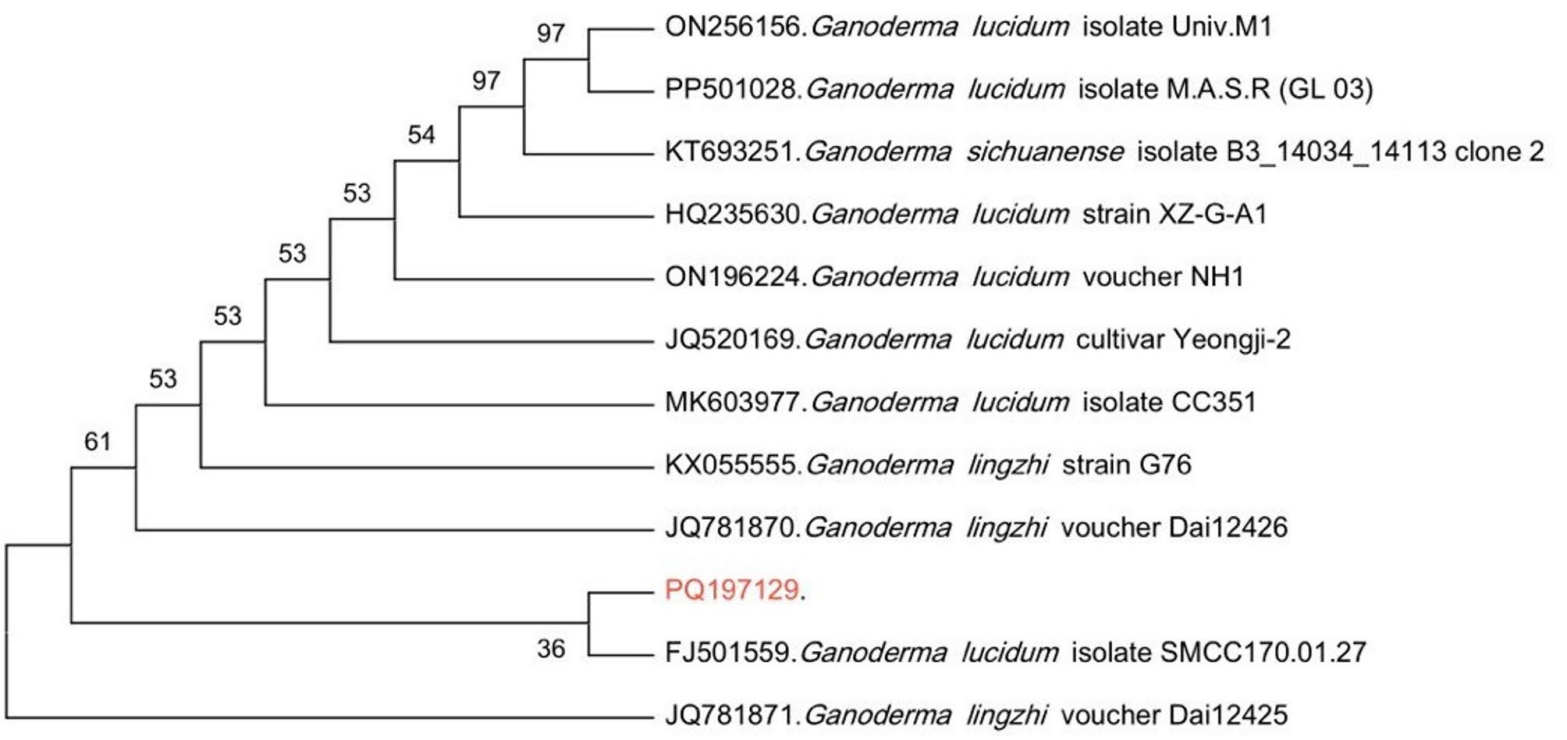
Phylogenetic tree of fungal sample based on ITS-1 and ITS-4 sequence data

The fungal isolate was identified as *G. lucidum* based on BLASTn findings, the sequence (Fig. S6) was deposited to GenBank® with accession code PQ197129. The Phylogenetic tree was constructed using MEGA 12.0 software based on ITS-1 and ITS-4 sequence data (Fig. 2). Also, the strain was added to the World Data Centre for Microorganisms (WDCM) Culture Collection Ain Shams University (CCASU) under strain number CCASU-2025–F14.

### Cytotoxic activity of GLWE against vero cell lines

The prepared extract showed low cell toxicity against the normal Vero cell line (Fig. S1) with CC_50_ 5,237 ± 1.6 µg/ml.

### Evaluation of antiviral activity of GLWE

In-vitro antiviral effects of GLWE were evaluated against human Adv7 as well as HSV-1. with EC_50_ and selectivity index (SI) listed in Table 1. Despite the fact that research on *G. lucidum* fruiting body aqueous extract’s antiviral effect is scarce, the results demonstrated modest antiviral activity compared with that of cidofovir and acyclovir, coupled with relatively low toxicity for *G. lucidum*.

**Table 1:** Antiviral activities of *G. lucidum*

Virus		EC_50_ (µg/mL)	CC_50_ (µg/mL)	SI
Ad7	GLWE	607.1 ± 1.9	5,237 ± 1.6	8.62
	Cidofovir	19.13 ± 0.14	79.68 ± 0.12	4.16
HSV1	GLWE	493.8 ± 0.98	5,237 ± 1.6	10.6
	Acyclovir	4.74 ± 0.13	116.57 ± 0.23	24.59

All experiments were independently repeated three times (n = 3), and results are expressed as mean ± SD. GLWE; *Ganoderma lucidum* water extract, Ad7; adeno-7 virus, HSV1; herpes simplex virus type 1, EC_50_; 50% effective concentration, CC_50_; 50% cytotoxic concentration, SI; selectivity index (SI = CC_50_/EC_50_).

### Cytotoxic activities of GLWE against tumor cell lines

**Fig. 3:**
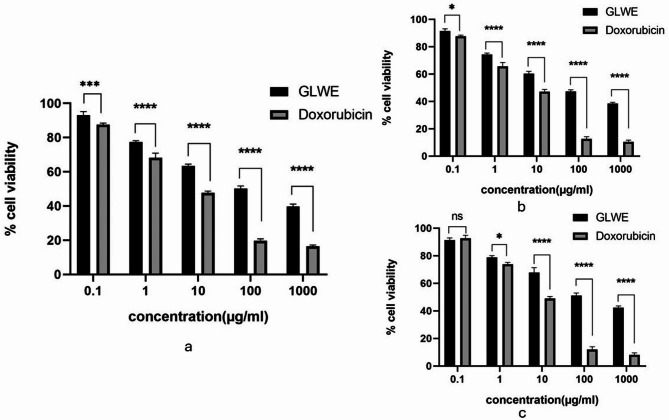
Assessment of cytotoxicity of GLWE using the MTT assay. HepG-2 (**a**), MCF-7 (**b**), and Caco2 cells were employed to estimate the anticancer effects of different concentrations of the fungal extract. Data represent mean ± SD from three independent experiments (n = 3). Statistical analysis was performed using two-way ANOVA followed by Bonferroni’s post hoc test for multiple comparisons. (*) p < 0.05, (**) p < 0.01, (***) p < 0.001, (****) p < 0.0001 and (ns) not significant p ˃ 0.05

The cytotoxic effect of GLWE and doxorubicin against selected cancer cell lines was conducted using 5 concentrations (0.1, 1, 10, 100, and 1000 µg/mL). According to the obtained findings, IC_50_ of GLWE against HepG2, Caco2, and MCF-7 were 138.2, 198.1, and 99.06 µg/mL, while doxorubicin recorded IC_50_ values of 6.91, 7.08 and 5.01 µg/mL, respectively (Fig. 3).

### Antibacterial and antifungal activities

**Fig. 4:**
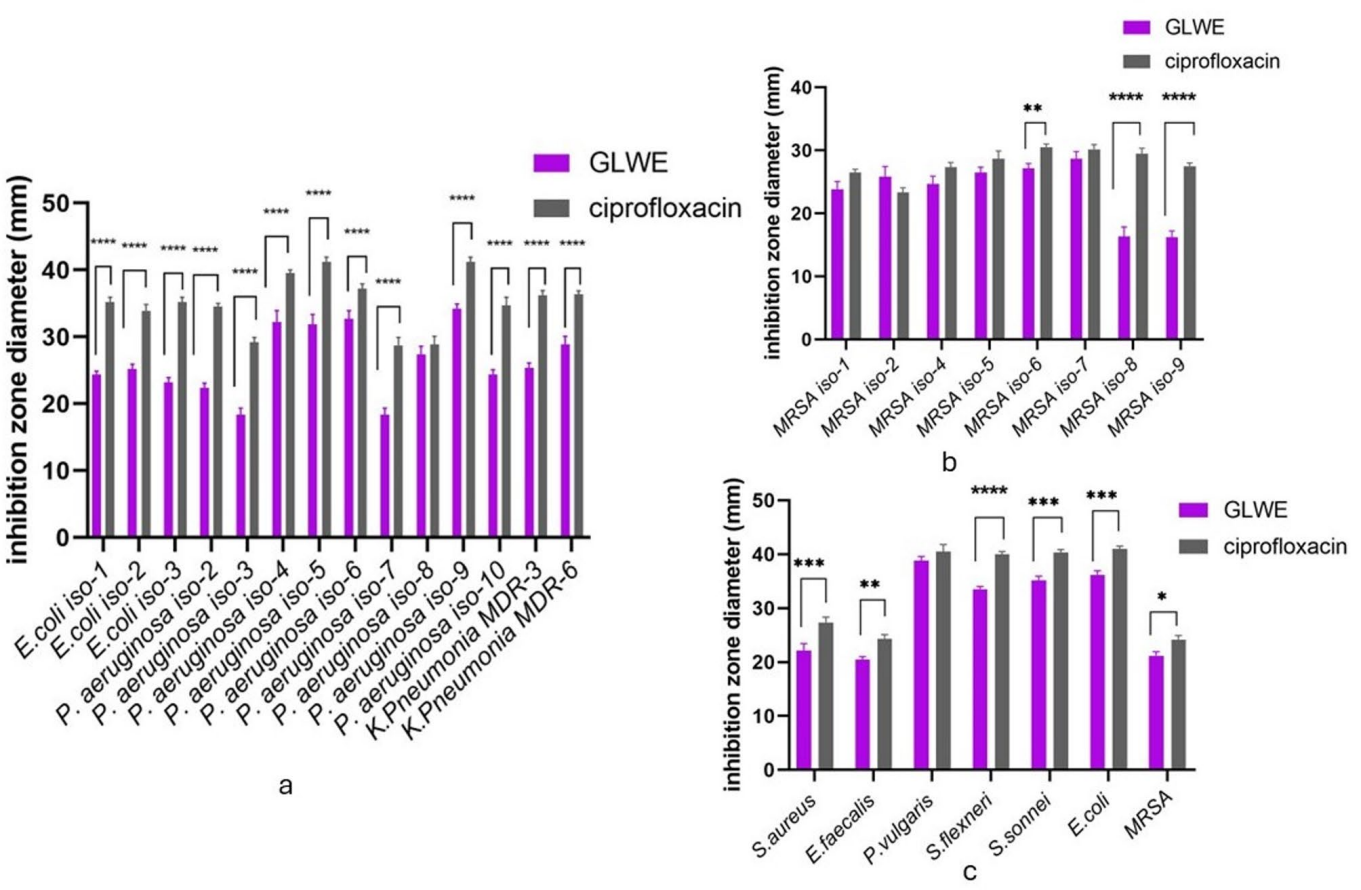
Assessment of the antibacterial efficacy of GLWE against **a** Clinical isolates of Gram-negative and multidrug-resistant strains, **b** Gram-positive MRSA strains, **c** ATCC standard strains. Data represent mean ± SD of three independent experiments per bacterial strain (n = 3 for each strain). Statistical comparisons across strains were performed using multiple paired t-test (n = 39). (*) p < 0.05, (**) p < 0.01, (***) p < 0.001 and (****) p < 0.0001

**Fig. 5:**
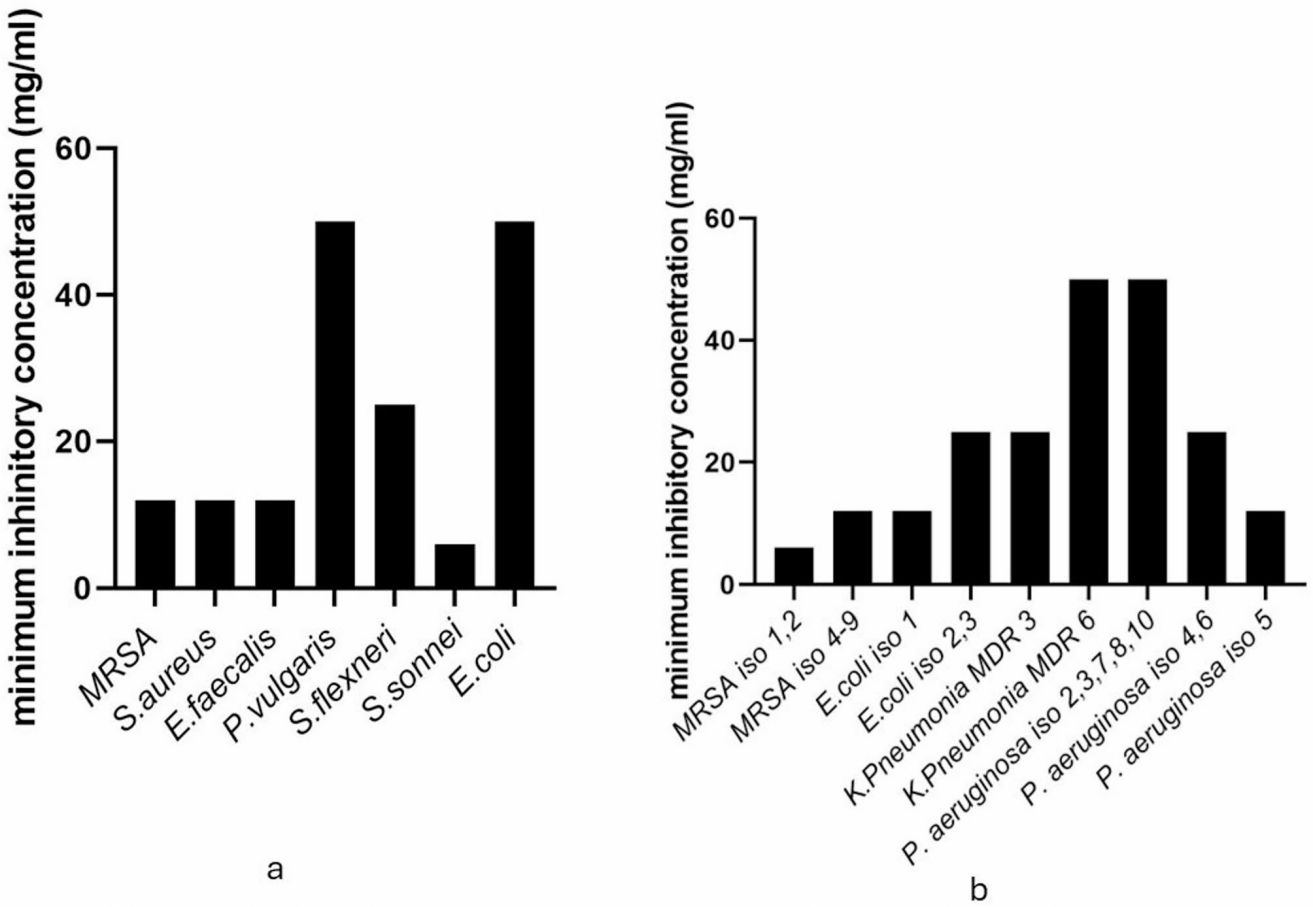
Minimum inhibitory concentration of GLWE against **a** ATCC standard strains, **b** Clinical isolates and multidrug-resistant strains

GLWE demonstrated promising antibacterial activity, predominately effective against the Gram-negative bacteria (*E. coli*, *S. flexneri*, *S. sonnei*, *P. aeruginosa*, and *P. vulgaris*) as well as Gram-positive strains (*S. aureus*, *E. faecalis*, and MRSA) (Fig. 4). When compared to ciprofloxacin (8 μg/mL), there was no significant difference (p ˃ 0.05) between the extract and control, as it produced inhibition zones nearly as large as ciprofloxacin’s, especially against *P. vulgaris* and MRSA strains (Table S2), while there was a significant difference between them against other strains (p < 0.05). However, no activity against all the tested unicellular (*C. albicans* ATCC 10231, *C. tropicalis* ATCC 13803), and filamentous fungi (*Aspergillus niger* RCMB 002005 and *Penicillium marneffeii* RCMB 001022). The minimum inhibitory concentrations of GLWE against bacterial strains are presented in Fig. 5**.**

### Antioxidant scavenging capacity of GLWE

Two distinct assessment techniques were used to examine antioxidant capacity using ascorbic acid as a standard antioxidant (Table 2). The ABTS test demonstrated that GLWE's capacity to scavenge free radicals was noticeably greater than the DPPH test’s.

**Table 2:** IC_50_ values for GLWE in the radical scavenging tests for DPPH and ABTS

IC_50_(μg/mL)		
	DPPH radical scavenging*	ABTS radical cation scavenging*
*G. lucidum*	23.69 ± 0.85^a^	14.79 ± 0.92^c^
Ascorbic acid	10.66 ± 0.89^b^	10.20 ± 0.74^b^

*All experiments were independently repeated three times (n = 3), and results are expressed as mean ± SD. Different letters indicate significance in differences (p < 0.05)

## Biochemical characterization of mushroom water extract

### Nutritional and functional phytoconstituents content

The biochemical characterization of the *G. lucidum* mushroom extract revealed notable variations in macromolecular content. The total content of carbohydrates was determined to be 52.11 ± 5.13 mg GluE/g, while the total phenolic content was 62.9 ± 2.20 mg GAE/g, indicating a relatively high presence of phenolic compounds. Total flavonoid content was quantified as 30.1 ± 3.23 mg QE/g, suggesting a moderate concentration of flavonoids. In contrast, total protein content was found to be 15.9 ± 5.13 mg BSAE/g, signifying a comparatively lower protein concentration. These results highlight the predominance of phenolic compounds in the aqueous extract, followed by carbohydrates and flavonoids, with proteins being the least abundant component.

### Vitamin content of GLWE

The concentration of water-soluble and fat-soluble vitamins was measured utilizing RP-HPLC, as presented in Table 3, with chromatograms of GLWE shown in Fig. S7 and those of standard vitamins shown in Fig. S8. Phylloquinone (vitamin K) was the most prevalent fat-soluble vitamin, while riboflavin (vitamin B2) was the most prevalent water-soluble vitamin. Additionally, fat-soluble vitamins E, A, and D, as well as water-soluble vitamins B1, B3, B5, B6, and C, were also detected.

**Table 3:** Vitamin content of GLWE

	Vitamin	Conc. (µg/mL)	Ret. time (min)
Water soluble Vitamins	Ascorbic acid (vit.C)	2.55 ± 0.39	2.0
	Riboflavin (vit. B2)	10.05 ± 0.06	3.0
	Pyridoxine (vit. B6)	9.41 ± 0.15	5.0
	Thiamine (vit. B1)	7.14 ± 0.03	6.0
	Niacinamide (vit. B3)	5.22 ± 0.02	7.0
	pantothenic acid (vit. B5)	6.87 ± 0.12	9.0
Fat soluble Vitamins	α Tocopherol (vit. E) Cholecalciferol (Vit. D) Phylloquinone (Vit.K) Retinol (vit. A)	3.43 ± 0.26 7.06 ± 0.09 11.29 ± 0.32 2.20 ± 0.05	5.0 6.2 8.0 9.0

Each value is presented as mean ± SD (n = 3).

### HPLC estimation of phenolics and flavonoids

HPLC was used to characterize the flavonoids and polyphenols in the GLWE (Fig. S9 and Table 4). According to the findings, 18 compounds were identified, including 11 phenolics and seven flavonoids. Gallic acid (66.17%) and chlorogenic acid (13.64%) were the main phenolic constituents. Flavonoid compounds were minimally present, with much higher area% for both Hesperetin and Quercetin (3.80, 3.64%), respectively.

**Table 4:** HPLC estimation of phenolics and flavonoids

RT	Compound	Type	Area	Area%
3.55	Gallic acid	Phenolic	700.66	66.17
4.23	Chlorogenic acid	Phenolic	144.41	13.64
4.56	Catechin	Flavonoid	3.61	0.34
5.48	Methyl gallate	Phenolic	14.45	1.37
5.82	Caffeic acid	Phenolic	24.73	2.34
6.35	Syringic acid	Phenolic	30.17	2.85
7.00	Rutin	Flavonoid	4.34	0.41
7.27	Ellagic acid	Phenolic	8.39	0.79
8.54	Coumaric acid	Phenolic	1.46	0.14
9.02	Vanillin	Phenolic	5.21	0.49
9.60	Ferulic acid	Phenolic	3.34	0.32
10.14	Naringenin	Flavonoid	2.86	0.27
12.16	Rosmarinic acid	Phenolic	3.22	0.30
15.32	Daidzein	Flavonoid	1.10	0.10
17.45	Quercetin	Flavonoid	38.56	3.64
18.99	Cinnamic acid	Phenolic	11.52	1.09
20.28	Kaempferol	Flavonoid	20.49	1.94
20.85	Hesperetin	Flavonoid	40.26	3.80

### LC–MS analysis for triterpenoids of GLWE

The analysis of triterpenoids in GLWE was initiated by employing negative ion ESI–MS to determine the molecular mass of the components through the detection of [M–H] ⁻ ions. The identification of these ions was further validated by acquiring MSⁿ spectra. The ESI–MS analysis was conducted in negative ion mode utilizing XEVO TQD triple quadrupole apparatus, and the resulting analytical data were processed using Masslynx software. The tentative identification revealed the presence of ten triterpene structures such as C_30_ or C_27_ lanostane listed according to their retention times (Table 5). The identified compounds showed fragmentations in good agreement with published data and compatible with suspected fragmentation patterns of the corresponding triterpenoid type (Table 5). The primary fragmentation pathways observed involved the loss of H₂O and CO₂ molecules. Additionally, the characteristic fragmentation patterns were marked by cleavages occurring at the C- and D-rings of the triterpenoid structure.

**Table 5:** Mass spectra of GLWE-identified triterpenoids. (This table is provided at the end of a text file as required)

LC–MS data	LC–MS data spectra	
Rt	7.278	
MS/MS (m/z)	551 [M-H_2_O]^−^	
Type	II	
Collision energy (V)	20	
Reference	(Biswal et al. 2022)	
Rt	9.872	
MS/MS (m/z)	473 [M– H– 41]^−^	
Type	V	
Collision energy (V)	20	
Reference	(Yang et al. 2007)	
Rt	9.967	
MS/MS (m/z)	553 [M-H_2_O]^−^ 511 [M-H_2_O-CH_2_ = CO]^−^	
Type	IV	
Collision energy (V)	35	
Reference	(Yang et al. 2007)	
Rt	10.424	
MS/MS (m/z)	493 [M-H_2_O]^−^ 449 [M-H_2_O-CO_2_]^−^	
Type	III	
Collision energy (V)	20	
Reference	(Yang et al. 2007)	
Rt	10.571	
MS/MS (m/z)	497 [M-H_2_O]^−^ 453 [M-H_2_O-CO_2_]^−^	
Type	III	
Collision energy (V)	35	
Reference	(Liu et al. 2011; Ćilerdžić et al. 2018)	
Rt	10.801	
MS/MS (m/z)	287	
Type	V	
Collision energy (V)	20	
Reference	(Yang et al. 2007)	
Rt	11.261	
MS/MS (m/z)	471 [M-CH_2_ = CO]^−^	
Type	V	
Collision energy (V)	20	
Reference	(Yang et al. 2007)	
Rt	12.306	
MS/MS (m/z)	481 [M-H_2_O]^−^	
Type	Derivative	
Collision energy (V)	20	
Reference	(Yang et al. 2007)	
Rt	19.546	
MS/MS (m/z)	483 [M-H_2_O]^−^	
Type	Derivative	
Collision energy (V)	20	
Reference	(Yang et al. 2007)	
Rt	28.558	
MS/MS (m/z)	395 [M-H_2_O-CH_2_ = CO]^−^	
Type	V	
Collision energy (V)	20	
Reference	(Yang et al. 2007)	

## Discussion

Mushrooms have received significant recognition as a prominent reservoir of bioactive substances utilized in the formulation of nutraceuticals together with medicinal products (Qi et al. 2023). Edible mushrooms' antiviral capabilities have been ascribed to aqueous extracts and generally associated with the availability of water-soluble polysaccharides (Zhang et al. 2022). *Pleurotus tuber-regium* β glucans show antiviral properties towards HSV-I (Zhang et al. 2004). Lentinan a β-D-glucan isolated from the fruiting body of shiitake mushroom (*Lentinula edodes*) has been demonstrated to have strong anti-HSV-I activity as well, according to Ali et al. (2003). Other compounds may potentially be responsible for antiviral action. For instance, two phenolic compounds that were isolated from *Inonotus hispidus* fruiting bodies were discovered to possess remarkable antiviral properties against influenza viruses.

Water was selected as the extraction solvent due to its safety, environmental sustainability, and its traditional use in the preparation of mushroom-based remedies. As a polar solvent, water is particularly effective in extracting hydrophilic bioactive compounds such as polysaccharides, phenolics, and other water-soluble metabolites that are commonly found in medicinal mushrooms and are known to contribute to antioxidant and antimicrobial activities. Additionally, the use of water aligns with green extraction principles and supports the potential application of the extract in food and pharmaceutical contexts (Lazić et al. 2024).

According to Zubair et al. 2021 selectivity index (SI) is commonly used parameter for evaluating the safety profile of a compound as an antiviral agent. SI quantifies the gap between the cytotoxic and antiviral activity. Higher SI values indicate higher efficacy and safety of drug use. The present study revealed that GLWE had mild antiviral properties against HSV-1 and HAdv7 with EC_50_ values of 493.8 ± 0.98 and 607.1 ± 1.9 µg/mL, respectively, and low normal cell cytotoxicity (CC_50_ 5237 ± 1.6 µg/mL), making the mushroom extract selective for targeting infected cells rather than normal ones with SI values of 10.6 and 8.62. However, compared to positive controls acyclovir (SI = 24.59) and cidofovir (SI = 4.16), the inhibitory activity was significantly different. The HPLC analysis showed that one of the primary ingredients of GLWE was chlorogenic acid, and that these acids, together with other phenolics and flavonoids, had a beneficial effect against HSV (Prinsloo et al. 2018).

Various bioactive components procured from mushrooms, including peptide RC28, polysaccharides, proteoglycans, sulfated polysaccharides, and triterpenoids (such as lucaldehyde B, ganoderone A, and ganodermadiol), have demonstrated significant antiviral effects (Mothana et al. 2003; Niedermeyer et al. 2005). Several phases of the viral replication cycle can be targeted by these substances. Notably, it has been discovered that peptide RC28’s antiviral efficacy against HSV is on par with that of ganciclovir, a proven antiviral medication. (Seo and Choi 2021). In addition to the in vitro studies, the in vivo studies have also demonstrated strong antiviral activity of peptide RC28 along with sulfated polysaccharide derived from mushrooms against the herpes simplex virus. These results point to their potential as viable therapeutic choices for the management of viral infections.

Recent investigations have further illuminated that polysaccharides isolated from *G. lucidum* have exhibited substantial antioxidant, anti-inflammatory, and immunomodulatory properties, primarily through the modulation of the NF-κB and MAPK signaling pathways, in addition to the activation of the Nrf2/Keap1 axis, which is integral to cellular defense mechanisms against oxidative stress. Triterpenoids, particularly ganoderic acids, have been demonstrated to exert anticancer effects by inducing apoptosis via mitochondrial pathways, impeding metastasis through the suppression of MMP-9 and IL-8, and orchestrating pivotal signaling cascades such as PI3K/Akt and JNK/p38 MAPK (Aslaminabad et al. 2022; Cancemi et al. 2024). Moreover, *G. lucidum* displays neuroprotective characteristics, with its bioactive compounds influencing neuroinflammation and oxidative stress, thus providing potential therapeutic pathways for neurodegenerative disorders (Lian et al. 2024).

The GLWE showed a moderate growth inhibition on hepatocellular carcinoma, colorectal adenocarcinoma, and breast adenocarcinoma cell lines, with the greatest inhibition against MCF-7, followed by HepG2 and finally Caco2 with IC_50_ (µg/mL) values 99.06, 138.2 and 198.1, respectively. GLWE showed a somewhat greater drop in MCF-7 cell viability, but a roughly proportional decline in HepG2, Caco2, and MCF-7 cell viability.

While the precise antiproliferative mechanisms of polysaccharides on in vitro cytotoxicity remain not fully elucidated, according to previous studies, incubating polysaccharides with cancer cell lines might cause notable changes in intracellular signaling pathways. These changes may subsequently trigger cell cycle arrest as well as apoptosis, thereby providing a plausible explanation for the observed antiproliferative action of polysaccharides in vitro (Wang et al. 2002). *Inonotus obliquus* mushroom aqueous extract has been documented to demonstrate notable antitumor effect against colon cancer HT-29 cells. The primary mechanism behind this anticancer action is the activation of apoptosis, leading to the targeted destruction of cancerous cells and the suppression of cellular proliferation. Extensive research has underscored the anticancer potential of *Inonotus obliquus*. Notably, sclerotia extracts obtained from this mushroom have been found to effectively inhibit cancer cell proliferation and disrupt protein synthesis (Lee et al. 2009). Furthermore, A tetraprenylphenol substance called suillin was extracted from *Suillus placidus* has been identified as a selective agent capable of targeting and eliminating human liver cancer cells. adding to its antiproliferative properties, suillin has been found to induce apoptotic cell death in HepG2 cells, further highlighting its potential as a promising anticancer therapy (Liu et al. 2009).

The antibacterial efficacy of some mushroom extracts against Gram-positive bacteria has been documented. The world's most farmed fungus, *Agaricus bisporus*, deserves special attention. For *Bacillus subtilis*, its methanolic extracts showed MIC 5 µg/mL, which is even lower than that of the common ampicillin, whose MIC is 12.5 µg/mL (Barros et al. 2008), and even demonstrated efficacy against *Staphylococcus aureus, Staphylococcus epidermidis, Micrococcus luteus, Micrococcus flavus,* and *Bacillus cereus* (Tambekar et al. 2006; Ozen et al. 2011; Öztürk et al. 2011). *Armillaria mellea* mycelium ethanolic extracts presented antibacterial action against *Sarcina lutea*, but no effect against other Gram positive bacteria was detected (Kalyoncu et al. 2010).

According to Ferreira et al. (2012), mushroom extracts and isolated components exhibit better antibacterial efficacy against Gram-positive compared to Gram-negative bacteria. However, our findings indicate that GLWE exhibits remarkable antibacterial efficacy against Gram-negative bacteria, with the largest inhibition zone diameter of 38 mm observed for *P. vulgaris* ATCC 49132. This enhanced activity may be ascribed to the existence of 27 identified bioactives, including 11 polyphenols, 7 flavonoids, in addition to 9 triterpenoids as previously discussed by Özkütük 2022; Lima et al. 2016 and Chen et al. 2023.

The World Health Organization has designated Gram-positive MRSA, in addition to Gram-negative *E. coli* as critical priority pathogens due to their antibiotic resistance, necessitating urgent intervention. The findings of this research elucidate that the fruit body extract of *G. lucidum* exhibits a possible activity against Gram-positive and negative bacteria as well as pathogenic strains of MRSA and certain multidrug-resistant (MDR) strains of *K. pneumoniae*.

Natural extracts often exhibit antimicrobial effects with a broader therapeutic window and minimal side effects compared to synthetic antibiotics like ciprofloxacin, which are associated with known adverse reactions. Thus, while direct zone size comparisons may not be quantitatively meaningful, the qualitative observation of inhibition is valuable and supports the potential of this natural product for further fractionation and bioactivity-guided isolation (Chen et al. 2024).

As highlighted by Priya et al. (2021), The variation in free radical scavenging efficacy between the ABTS and DPPH techniques is attributed to the mechanisms, indicating a different interaction dynamic between the antioxidants and the radical species. The mechanisms are either sequential proton loss electron transfer (DPPH) or hydrogen atom transfer (ABTS) (Gasmi et al. 2022). The ABTS showed that GLWE antioxidant capacities were slightly lower than that of standard ascorbic acid, indicating the ability to hydrogen atom transfer, which in accordance, has valuable role in heterogeneous Fenton oxidation processes. In these processes, ABTS enhances the degradation of pharmaceuticals by acting as a redox mediator, facilitating the cycling of reactive species (Li et al. 2024). This activity may be explained by reasonable polyphenolic and flavonoid compounds (Yang et al. 2008) (Table 4) and further confirmed by the tentatively identification of 25 antioxidant compounds, of which 11 polyphenols, 7 flavonoids and 6 triterpenoids (Skroza et al. 2022; Girsang et al. 2020a).

The IC_50_ values for *G. lucidum* were determined to be 14.79 ± 0.92 µg/mL and 23.69 ± 0.85 µg/mL for the ABTS and DPPH assays, respectively. Antioxidant potential of the test extract is likely attributable to the existence of diverse bioactive compounds. Notably, the GLWE contains a significant amount of vitamin C (2.55 µg/mL). Antioxidants, such as vitamins C and E, function as non-enzymatic scavengers of free radicals. vitamin C, being water-soluble, is particularly effective in combating free radical damage both intracellularly and extracellularly (Salehi et al. 2018). While vitamin C is widely recognized for its antioxidant activity, recent studies have also proposed non-calcemic roles for vitamin D, including its potential antioxidant effects. These effects may be mediated by nitric oxide synthase enzyme inhibition or by modulating glutamate levels (Elhusseiny et al. 2021). Additionally, mushrooms contain catechin, a compound associated with antioxidant activity. Catechins are believed to postulated to manifest their effects via mechanisms such as chain-breaking and the suppression of lipid peroxidation in low-density lipoprotein (LDL) (Lambert and Elias 2010).

Both nutritional and functional constituents were included in the biochemical investigation to highlight the importance of GLWE in a typical human diet and to clarify its beneficial effects. The detection of a diminished protein concentration (15.99 mg/g) in conjunction with a relatively low carbohydrate level (52.11 mg/g) renders the mushroom water extract a valuable nutritional resource for individuals suffering from obesity, diabetes as well as chronic kidney diseases as previously documented by Ahmad et al. (2021), who proposed *G. lucidum* for diabetics and weight-watchers (Rhee et al. 2018). However, in comparison with the findings of S. T. Chang & Buswell, (1996) as well as Kozarski et al. (2012), less carbohydrate and protein content were found in our study. The reasonable concentration of vitamins, mainly B2 and B6 (10.05, 9.41 ug/mL, respectively) raise the mushroom water extract nutritional and pharmacological value where an adequate intake of vitamins B2 and B6 plays a role in the cellular metabolism and exerts potent antioxidant activities neutralizing reactive oxygen species that may cause deleterious effects like cell degeneration in the body. They can also improve certain neurological conditions in the eyes, mucosa, and skin (Sechi et al. 2016; Theodosis-Nobelos and Rekka 2024). In moderation, vitamins D and K (7.06, 11.29 μg/ml, respectively) provide substantial pharmacological and nutritional advantages and play a crucial role in various physiological processes, including blood coagulation, bone health, and cardiovascular function (Gasmi et al. 2022). The observed activities, including antimicrobial, cytotoxicity, antiviral, and antioxidant, were explained by quantification of total polyphenols and flavonoids along with their nomination using HPLC in addition to LC/MS tentative identification of mushroom triterpenoids. The reasonable contents of phenolics and flavonoids (62.9 and 30.1 mg/g, respectively) emphasize the valuable activities in a comparable way to previously investigated strains and extracts of the mushroom. The total polyphenols and flavonoid compounds were previously mentioned in several published studies. Total phenolic content was determined to range between 9 and 63 mg GAE/g, while total content of flavonoids ranges from 4 to 32 mg QE/g (Mohsin et al. 2011; Abdullah et al. 2012; Keypour et al. 2019). The identified phenolics (11 compounds), flavonoids (7 compounds), and triterpenoids (10 compounds) have previously been demonstrated to mediate the observed bioactivities as indicated in the literature (Ivanov et al. 2022b; Cör Andrejč et al. 2022; Ninfali et al. 2020; Veiko et al. 2023). Catechin, gallic acid and chlorogenic acid, which have been directly linked to the antioxidant efficacy of natural extracts, were detected in the mushroom in adequate amounts (Maeda et al. 2018). Similarly, Butkhup et al. (2018) proclaimed the existance of catechins in more than 23 different edible mushrooms. Unlike Yildiz et al. (2015), who reported that *G. lucidum* does not have gallic acid, our findings revealed that GLWE has high concentrations of gallic acid. This variance could result from the strains' varying rates of growth. Additionally, the growing medium, harvesting stage, and duration between harvest and measuring techniques may all influence how nutritionally stable the different strains of mushrooms are. (Chang and Buswell 1996).

In summary, the outcomes of this study reveal that wild *G. lucidum* specimens collected from Egypt exhibit antiviral, antitumor, antibacterial and antioxidant activities. Triterpenoids, polyphenols, flavonoids, and polysaccharides are among the many different types of bioactive substances that are thought to be responsible for these effects. The broad spectrum of these compounds provides comprehensive protection, as each compound operates through distinct mechanisms, creating a synergistic effect. The ease and simplicity of extracting such marvelous compounds by only macerating the mushroom in water make it a modest and applicable treatment for various health conditions. Consequently, incorporating *G. lucidum* mushrooms into the human diet may serve as a potential strategy to mitigate oxidative stress, minimize the risk of tumors, and protect against certain microbial and viral infections. However, the relationship between the in vitro findings and their relevance to in vivo conditions in healthy individuals remains uncertain, and the lack of mechanistic validation further underscores the need for additional research evaluating a broader range of biomarkers in in vivo settings.

## Electronic supplementary material

Below is the link to the electronic supplementary material.

Supplementary Material 1.

## Data Availability

All data collected and examined in the present investigation are detailed within the published manuscript and its accompanying supplementary file. The genomic DNA of the isolated fungus was subjected to sequencing, assembly, and annotation prior to its submission to the NCBI GenBank® repository, assigned the nucleotide accession code PQ197129.

## Abbreviations

CBBG-250: Coomassie Brilliant Blue G-250
CCID_50_: Cell culture infectious dose 50%
CPE: Cytopathic effect
E-MEM: Eagle’s minimum essential medium
ESI-MS: Electrospray ionization mass spectrometry
GAE: Gallic acid equivalent
GLWE: *Ganoderma lucidum* Water extract
GluE: Glucose equivalent
ITS: Internal transcribed spacer
LC-MS: Liquid chromatography mass spectroscopy
MDR: Multidrug-resistant
MIC: Minimum inhibitory concentration
MRSA: Methicillin-resistant *S. aureus*
PBS: Phosphate buffer saline
SI: Selectivity index
QE: Quercetin equivalent
