# Supplementary material for: Unlocking Reishi’s secrets: nutritional and medicinal traits of Ganoderma lucidum isolated from tree bark in Egypt
Source: AMB Express. 2025 Jul 12;15:104. doi: 10.1186/s13568-025-01905-6 (PMC12255602; doi:10.1186/s13568-025-01905-6)
Supplement: Supplementary file 1 — Supplementary Material 1. [file 13568_2025_1905_MOESM1_ESM.docx]

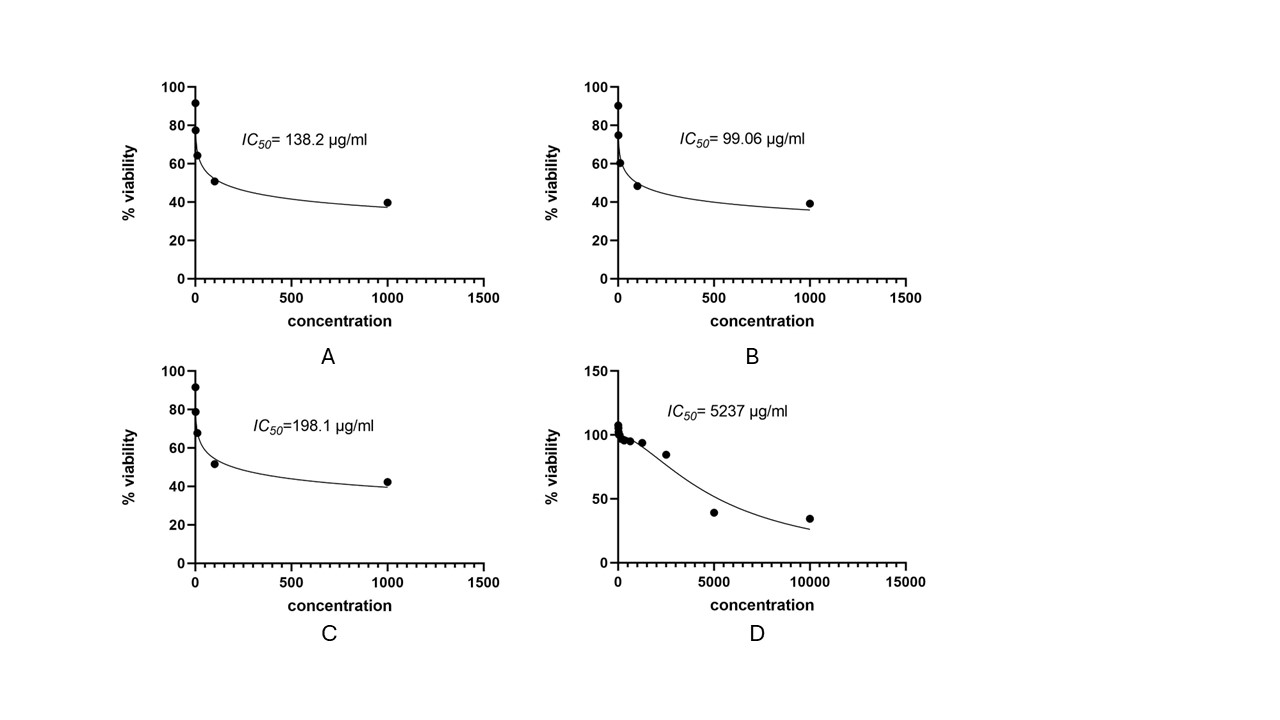


**Fig. S1** Dose-response curves of GLWE against (**A**) HepG2, (**B**) MCF-7, (**C**) Caco2 and (**D**) Vero cell lines.


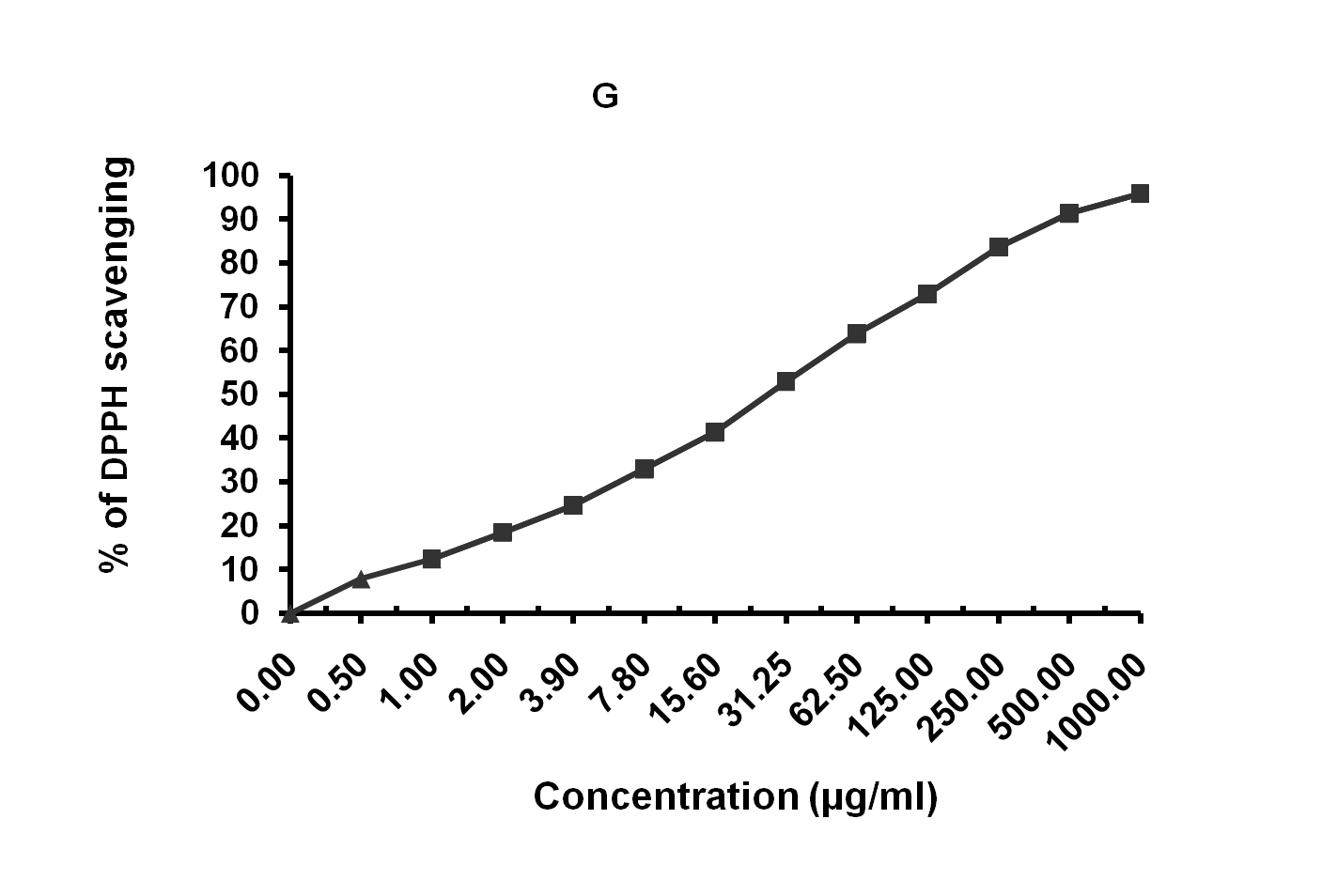


IC_50_= 23.69±0.85 µg/ml.

**Fig. S2** DPPH dose-response curve and IC50 of the GLWE


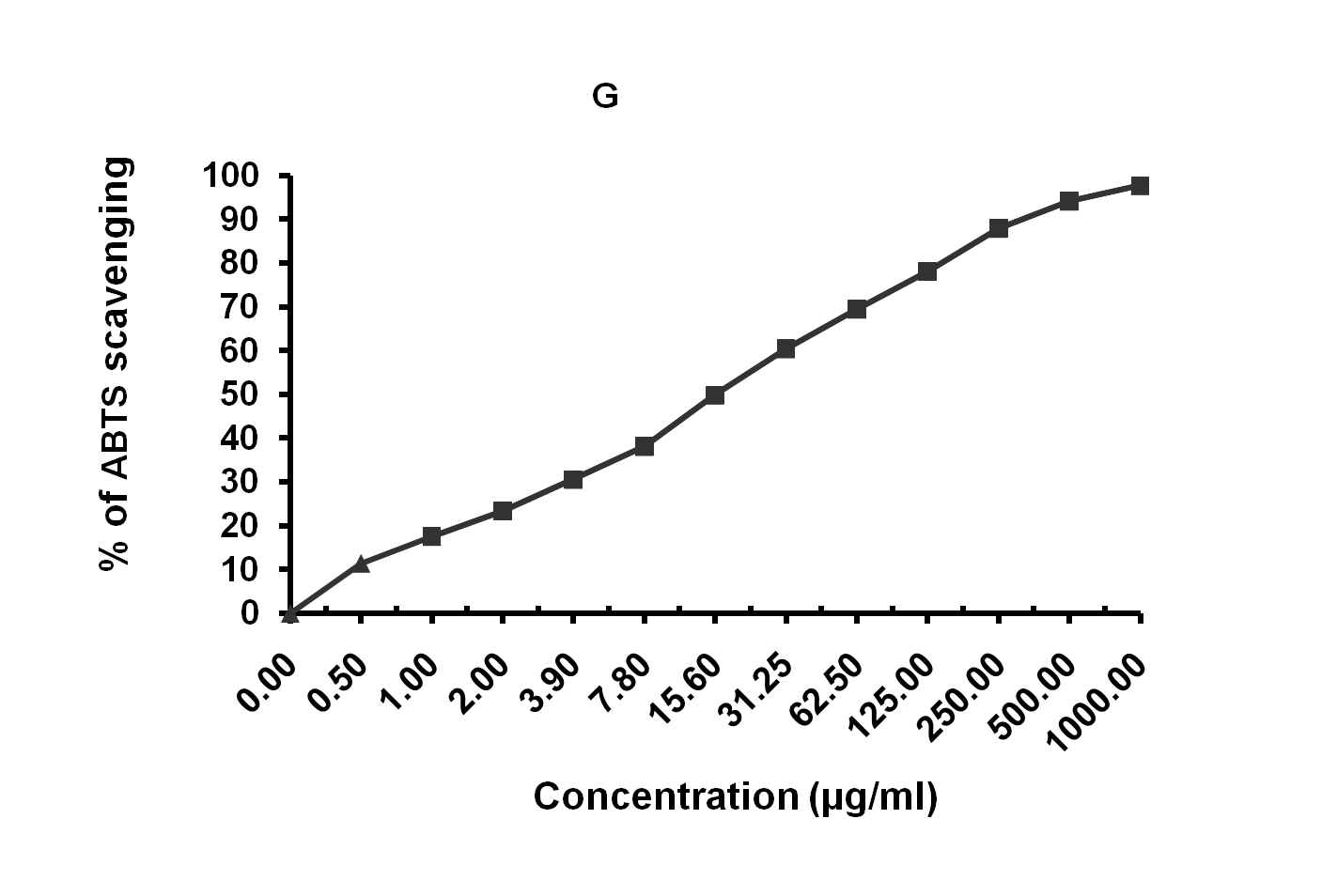


IC50= 14.79±0.92 µg/ml.

**Fig. S3** ABTS dose-response curve and IC50 of the GLWE


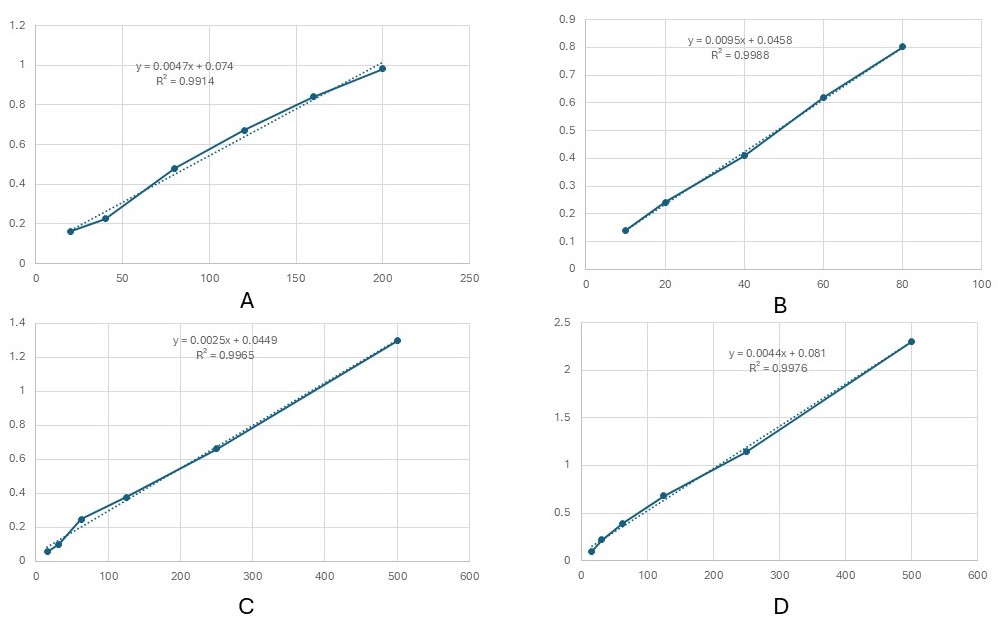


**Fig. S4** Standard calibration curves of glucose (**A**), bovine serum albumin (**B**), gallic acid (**C**), and quercetin (**D**).


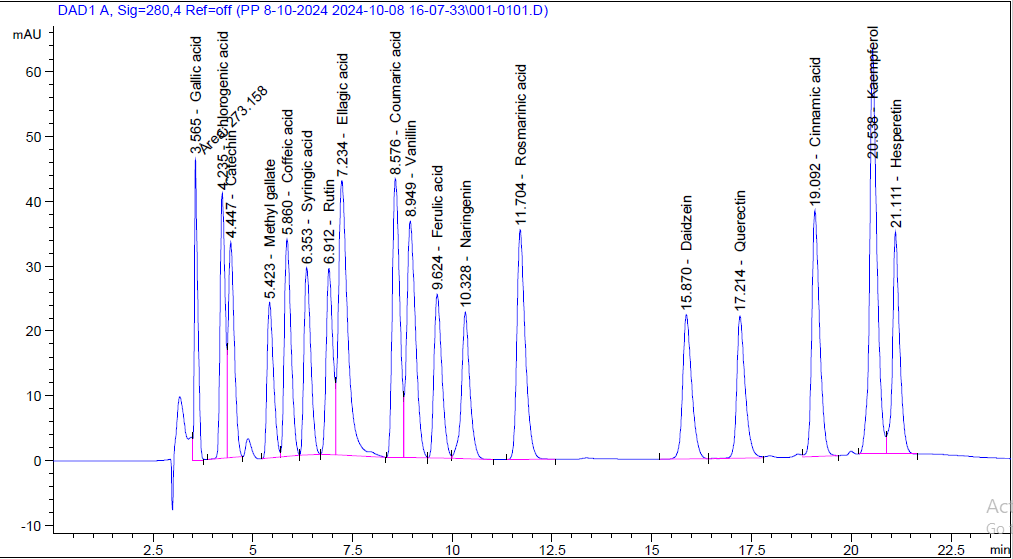


**Fig. S5** HPLC chromatogram of standard polyphenols and flavonoids.


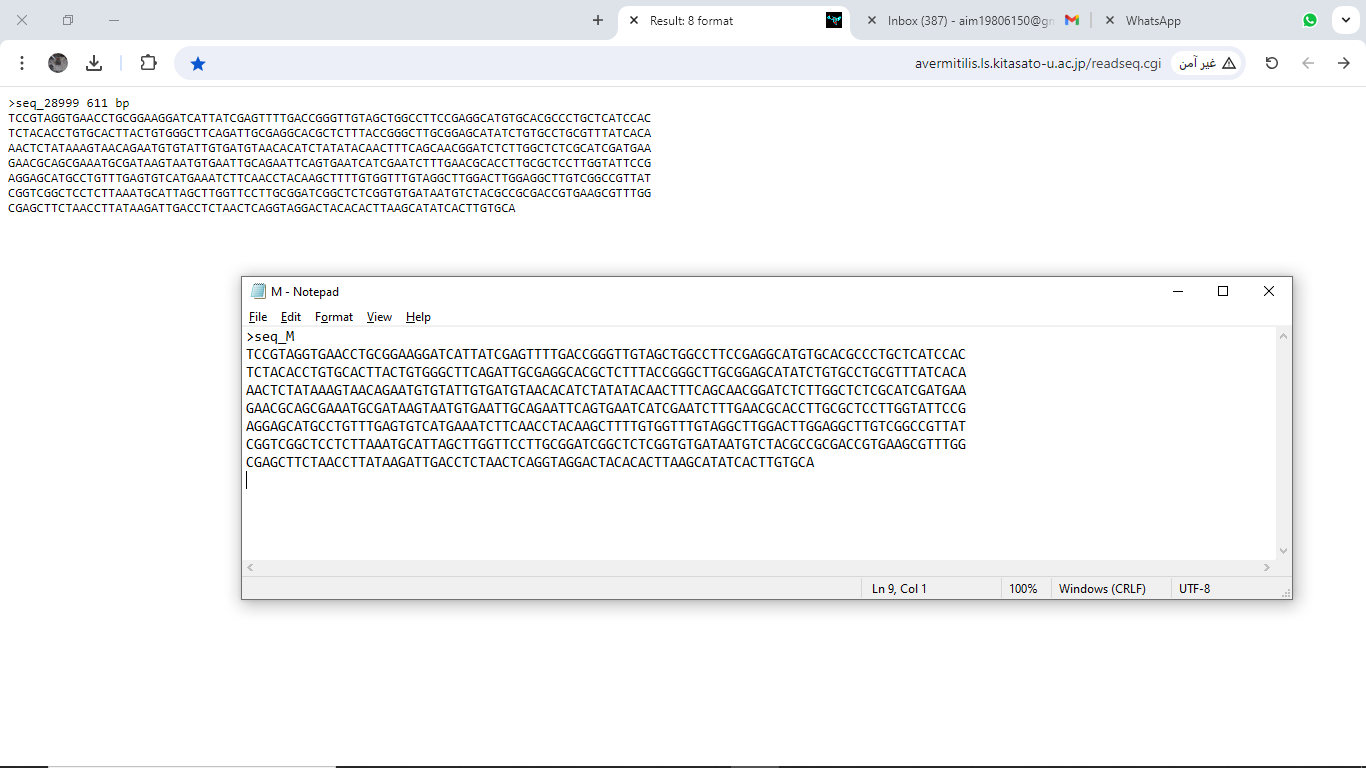


**Fig. S6** DNA sequence of *G. lucidum* sample.


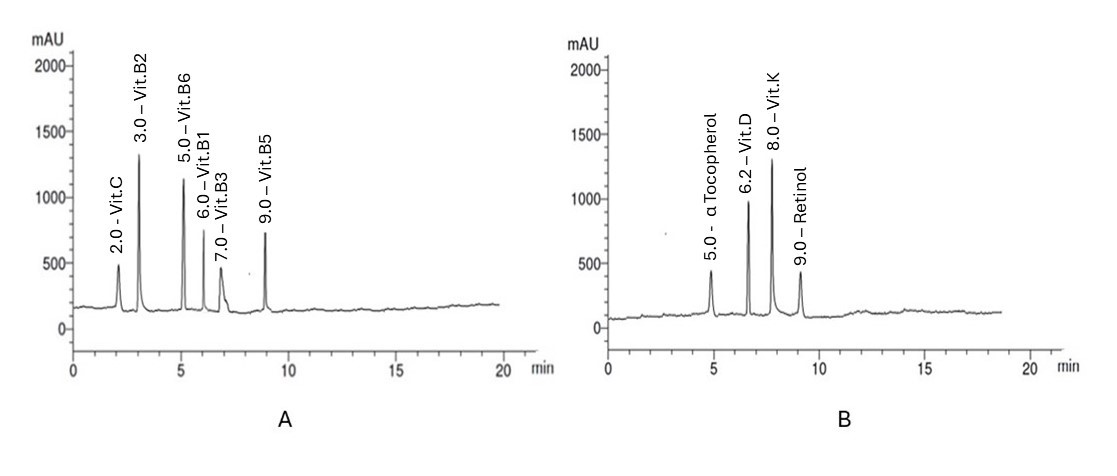


**Fig. S7** HPLC chromatograms of GLWE (**A**) water-soluble vitamins (**B**) fat-soluble vitamins


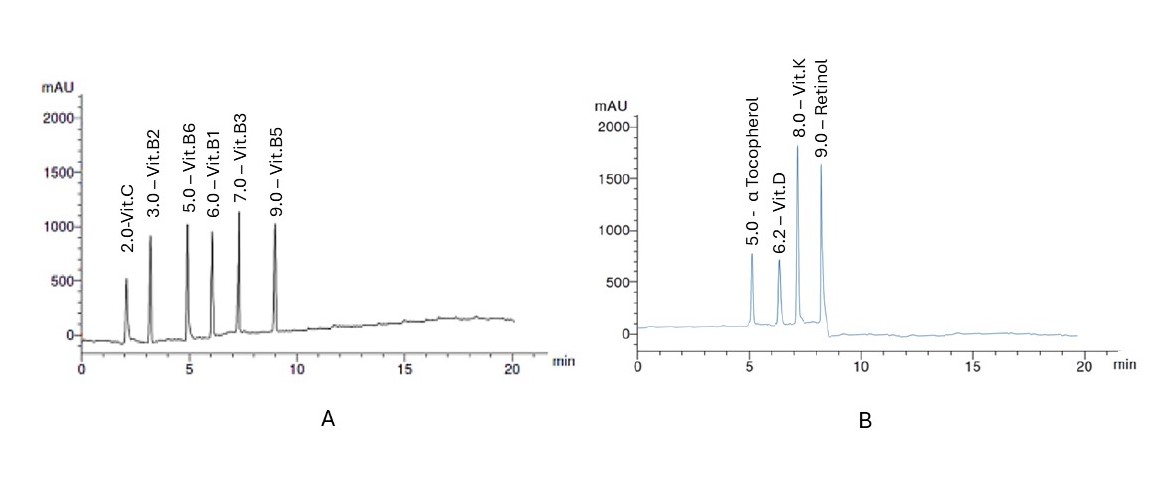


**Fig. S8** HPLC chromatograms of standard (**A**) water-soluble vitamins (**B**) fat-soluble vitamins


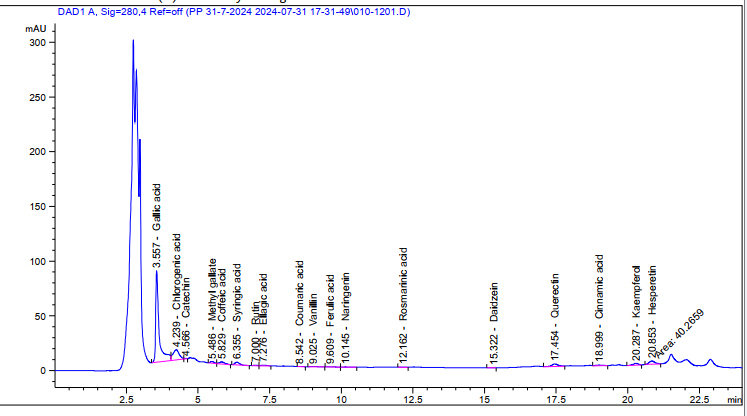


**Fig. S9** HPLC chromatogram of phenolics and flavonoids in mushroom water extract

**Table S1** data analysis for DNA ladder and *G. lucidum* sample.


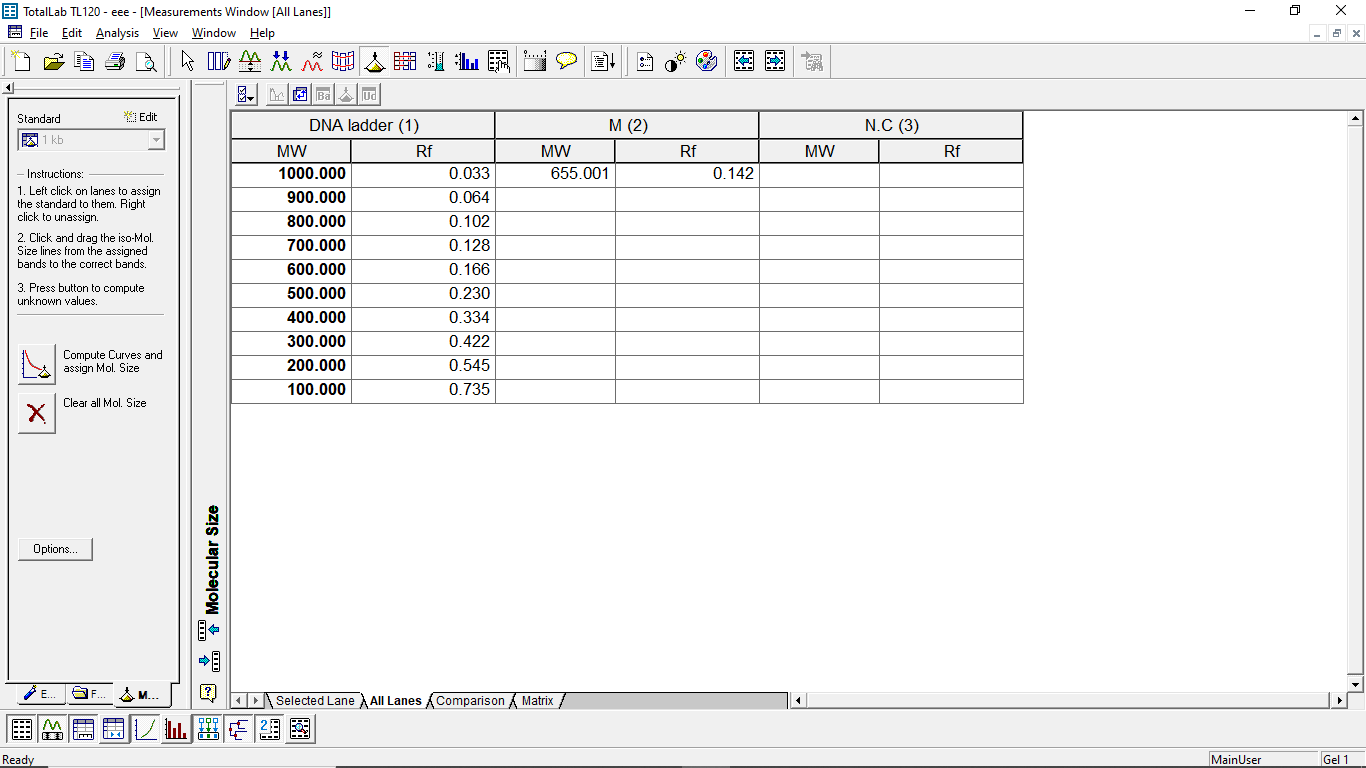


**Table S2** Antimicrobial activities of GLWE

|  | | Strains code | | GLWE  (50mg/mL) | ciprofloxacin  (8μg/mL) | | | MIC | | | |
| --- | --- | --- | --- | --- | --- | --- | --- | --- | --- | --- | --- |
|  |  |  |  |  |  |  |  | Linezolid  (μg/mL) | | | GLWE (mg/mL) |
| *Gram- positive* | | MRSA (ATCC 25923) | | 21.17±0.77 | 24.17±0.77 | | | 2 | | | 12.5 |
|  |  | MRSA iso-1 | 23.84±1.26 | | 26.5±0.5 | | 1 | | | 6.25 | |
|  |  | MRSA iso-2 | 25.84±1.61 | | 23.34±0.77 | | 1 | | | 6.25 | |
|  |  | MRSA iso-4 | 24.67±1.26 | | 27.34±0.77 | | | 4 | | | 12.5 |
|  |  | MRSA iso-5 | 26.5±0.87 | | 28.67±1.26 | | | 2 | | | 12.5 |
|  |  | MRSA iso-6 | 27.17±0.77 | | 30.5±0.5 | | | 4 | | | 12.5 |
|  |  | MRSA iso-7 | 28.67±1.16 | | 30.17±0.77 | | | 4 | | | 12.5 |
|  |  | MRSA iso-8 | 16.34±1.53 | | 29.5±0.87 | | | 4 | | | 12.5 |
|  |  | MRSA iso-9 | 16.17±1.05 | | 27.5±0.5 | | | 4 | | | 12.5 |
|  | *S.aureus (ATCC 43306)* | | 22.17±1.26 | | 27.34±1.05 | | | 2 | | | 12.5 |
|  | *E.faecalis (ATCC 19433)* | | | 20.5±0.5 | | 24.34±0.77 | | 2 | | | 12.5 |
|  |  | | |  | |  | | Ciprofloxacin  (μg/mL) | GLWE (mg/mL) | | |
| *Gram- negative* | *P.vulgaris (ATCC 49132)*  *S.flexneri (ATCC 12022)*  *S.sonnei (ATCC 25931)*  *E.coli (ATCC 25922)*  *E.coli iso-1*  *E.coli iso-2*  *E.coli iso-3*  *E.coli iso-4*  *E.coli iso-5*  *E.coli iso-6*  *K.Pneumonia (ATCC 700603)*  *K.Pneumonia MDR-1*  *K.Pneumonia MDR-2*  *K.Pneumonia MDR -3*  *K.Pneumonia MDR-4*  *K.Pneumonia MDR-5*  *K.Pneumonia MDR-6*  *P. aeruginosa (ATCC 27853)*  *P. aeruginosa iso-1*  *P. aeruginosa iso-2*  *P. aeruginosa iso-3*  *P. aeruginosa iso-4*  *P. aeruginosa iso-5*  *P. aeruginosa iso-6*  *P. aeruginosa iso-7*  *P. aeruginosa iso-8*  *P. aeruginosa iso-9*  *P. aeruginosa iso-10* | | | 38.84±0.77  33.5±0.5  35.17±0.77  36.17±0.77  24.34±0.58  25.17±0.77  23.17±0.77  R  R  R  R  R  R  25.34±0.77  R  R  28.84±1.26  R  R  22.34±0.77  18.34±1.05  32.17±1.76  31.84±1.53  32.67±1.26  18.34±1.05  27.34±1.26  34.17±0.77  24.34±0.77 | 40.5±1.33  40±0.5  40.34±0.58  41±0.5  35.17±0.77  33.84±1.05  35.17±0.77  R  R  R  R  R  R  36.17±0.77  R  R  36.34±0.58  R  R  34.5±0.5  29.17±0.77  39.5±0.5  41.17±0.77  37.17±0.77  28.67±1.26  28.84±1.26  41.17±0.77  34.67±1.26 | | | 0.031  0.015  0.01  0.004  1  2  2  -  -  -  -  -  -  2  -  -  2  -  -  2  4  0.5  1  1  2  2  0.5  4 | | | 50  25  6.25  50  12.5  25  25  -  -  -  -  -  -  25  -  -  50  -  -  50  50  25  12.5  25  50  50  12.5  50 |

The mean width of inhibition zones in millimeters (±SD) was used to express the antibacterial activity results. Iso: clinical isolates. MDR: multidrug-resistant. R: resistant
